# Automatic Mapping Extraction from Multiecho T2-Star Weighted Magnetic Resonance Images for Improving Morphological Evaluations in Human Brain

**DOI:** 10.1155/2013/202309

**Published:** 2013-11-27

**Authors:** Shaode Yu, Shibin Wu, Yaoqin Xie

**Affiliations:** ^1^Shenzhen Institutes of Advanced Technology, Chinese Academy of Science, China; ^2^Shenzhen Key Lab for Low-Cost Healthcare, 1068 Xueyuan Avenue, Shenzhen University Town, Shenzhen, China

## Abstract

Mapping extraction is useful in medical image analysis. Similarity coefficient mapping (SCM) replaced signal response to time course in tissue similarity mapping with signal response to TE changes in multiecho T2-star weighted magnetic resonance imaging without contrast agent. Since different tissues are with different sensitivities to reference signals, a new algorithm is proposed by adding a sensitivity index to SCM. It generates two mappings. One measures relative signal strength (SSM) and the other depicts fluctuation magnitude (FMM). Meanwhile, the new method is adaptive to generate a proper reference signal by maximizing the sum of contrast index (CI) from SSM and FMM without manual delineation. Based on four groups of images from multiecho T2-star weighted magnetic resonance imaging, the capacity of SSM and FMM in enhancing image contrast and morphological evaluation is validated. Average contrast improvement index (CII) of SSM is 1.57, 1.38, 1.34, and 1.41. Average CII of FMM is 2.42, 2.30, 2.24, and 2.35. Visual analysis of regions of interest demonstrates that SSM and FMM show better morphological structures than original images, T2-star mapping and SCM. These extracted mappings can be further applied in information fusion, signal investigation, and tissue segmentation.

## 1. Introduction

As a routine examination technique, magnetic resonance imaging (MRI) has been extensively used in clinical diagnosis. Pixel intensities on conventional MR images are dependent on a complex mix of proton density (PD), longitudinal relaxation time (T1), and transverse relaxation time (T2) or T2-star relaxation time based on the initial scan setting [1–3]. Many types of MRI have been invented to reflect physical and physiological properties, such as T2-star weighted MRI and susceptibility weighted imaging (SWI) [4, 5]. Among these techniques, T2-star weighted MRI has been widely used to reveal functional and morphological characteristics by taking advantage of differences in tissue properties [6–10]. As an essential modality, T2-star weighted MRI is capable of producing a large number of medical images by selecting optimal cross section and imaging parameters for specific emphasis. How to dig out valuable messages from a series of MR images is an important project for various applications.

Quantitative MRI (Q-MRI) is one way to extract tissue-intrinsic information from a series of MR images [6–18]. Conventional MRI focuses on qualitative visual assessment of anatomy and disease. It interprets anatomic changes when there is visibly detectable difference in signal intensities, while improper imaging and timings may dramatically distort image quality and mislead the diagnosis. In contrast, Q-MRI seeks to quantify fundamental biologic messages and MR-inducible tissue properties. Quantitative measurements are theoretically independent of experimental settings and absolute and comparable regardless of scanners, institutions and time points. Q-MRI provides information that is intrinsically more tissue specific and is consequently less dependent on subjective visual assessment. It becomes mainstream in current clinical practice but has not yet been in routine use in diagnosis.

Similarity mapping (SM) is another way to extract tissues with similar behavior from a series of medical images with injected contrast agent [19–22]. Similarity values based on dynamic images are determined by measuring signal correlations of the temporal behaviors between different pixels and a selected region of interest (ROI). Reference [19] adopted normalized cross-correlation to calculate signal similarity, and [20] used an autoregressive moving average model. Reference [21] extended these SM techniques to study oncological dynamic positron emission tomography (PET) images and disproved the effectiveness of cross-correlation and normalized cross-correlation in PET studies. Reference [22] proposed tissue similarity mapping (TSM) based on mean square error (MSE) between a reference ROI and all other pixels over measurements. TSM is able to estimate the relative cerebral blood volume map dependent only on the signal intensity time course and uncover potential existence of multiple sclerosis. Although contrast agents are commonly used drugs, there are still many open and serious questions regarding toxicity and hypersensitivity [23, 24].

SCM is proposed [25] by replacing signal response to time course in TSM with signal response to TE changes in multiecho T2-star weighted MR without contrast agent. SCM is able to improve image quality and morphological evaluation. Since different tissues are with different sensitivities to reference signals, an improved algorithm is proposed by adding signal sensitivity into the theory of SCM and obtains two mappings. To exclude uncertainties from manual delineation, noise, artifacts, and partial volume effects, the new method is adaptive to generate an optimal reference signal by maximizing the sum of CI from SSM and FMM.

## 2. Method

### 2.1. Modified Similarity Coefficient Mapping

Signal intensities on MR images are dependent on a complex mix of PD, T1, and T2-star based on initial parameter settings. As to multiecho T2-star weighted MR imaging sequence, signal intensities versus TE values are expressed as *I*_*i*_ = *W* × exp⁡(−TE/*T*_2_*), where *W* reflects a mix of PD, T1, and the gain of system. Here we assume that a series of MR images are acquired from n-echo T2-star weighted MRI sequence, and let *I* = {*I*_1_, *I*_2_,…, *I*_*n*_} be spatially registered with ascending TE values. For any pixel (*i*, *j*), there is a row vector of pixel intensities *V*_*ij*_ = {*V*_*ij*1_, *V*_*ij*2_,…, *V*_*ijn*_}. Then manually delineate a ROI and average these pixel intensities in it as a reference signal *R* = {*R*_1_, *R*_2_,…, *R*_*n*_}. Pixel intensity of (*i*, *j*) on TSM [22] is generated by a MSE between *V*_*ij*_ and *R*. When calculating relative cerebral blood volume map, TSM introduces a local blood volume *λ*. SCM interpreted *λ* as a similarity index. It is known that rough measurement of relative signal strength may omit to investigate signal differences to TE changes, so we added a fluctuation index *ξ* into MSE, and we have MSE_*ij*_ = (1/*n*)∑_*k*=1_^*n*^||*V*_*ijk*_−*λ*_*ij*_ *R*_*k*_−*ξ*_*ij*_||^2^. To minimize the value of MSE_*ij*_, let the partial derivative of the first order with respect to *λ* and *ξ* be equal to 0. Optimal *λ* and *ξ* are calculated in (1). All *λ*_*ij*_ and *ξ*_*ij*_ make up the two mappings, SSM and FMM

(1) $$\begin{array}{c} \lambda_{ij}=\frac{\bar{V_{ij}R}-\bar{V_{ij}}\bar{R}}{\bar{R^{2}}-{\bar{R}}^{2}},\\ \xi_{ij}=\bar{V_{ij}}-\lambda_{ij}\bar{R}. \end{array}$$

Operator ^“¯”^ denotes the calculation of average value. It is clear that the meaning behind our algorithm is a linear fitting problem from (1). Pixel intensities on SSM may range from 0 to +*∞*. Maximum value in SSM depends on the intensity level of signal from image series and the reference. If one signal is similar to the reference, its value on SSM will be around +1, and its value on FMM is close to 0. That means that our method is more stable than SCM with consideration of fluctuations in signal comparison.

### 2.2. Contrast Measurement Index

How to measure image contrast is often difficult, especially when these pixel intensities between images and mappings show different meanings. Contrast is usually defined as the difference in mean luminance between an object and its surroundings [26, 27]. Commonly used CI is a nonreference index which is the average value of the local contrast in image region. The local contrast in each region is measured in a local window

(2) $$\begin{array}{c} \text{CI}=\frac{m_{f}-m_{b}}{m_{f}+m_{b}}. \end{array}$$

In (2), *m*_*f*_ is the mean luminance value of the foreground and *m*_*b*_ is equal to the mean luminance value of background. In our experiments, the size of local window is 7 × 7. Based on CI values, we adopt a quantitative measurement of contrast improvement index (CII) [27] and CII = CI_map_/CI_ori_. Here, CI_map_ denotes CI value of the mapping and CI_ori_ is CI value of an original image. CII demonstrates improvement of image contrast in derived mappings compared to these original images.

### 2.3. Optimal Reference Signal Determination

The reference signal *R* is critical in real applications, while manual delineation may introduce errors and uncertainties. In addition, partial volume effects, artifacts, and noise are inevitable in MR images. Here we choose to automatically generate reference signal *R* with optimal weight *W* and T2-star by maximizing the sum of CI values from SSM and FMM.

**Figure 1:**
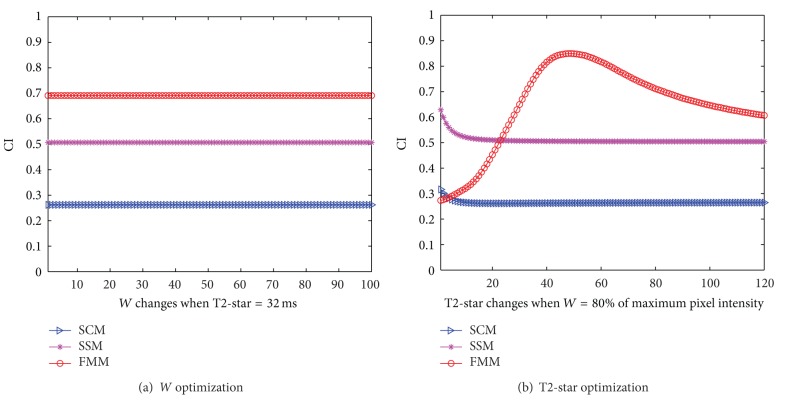
Optimal parameters determination of reference signal.

Taking one series of MR images, for instance, Figure 1(a) shows CI value with *W* varying from 1% to 100% of maximum intensity in original images and T2-star is set as equal to 32 ms. The CI values of SCM, SSM and FMM have no variations with respect to the *W* value of reference signal. It is easy to understand that changes of *W* have only influence on the scale of similarity strength. With T2-star varying from 1 ms to 120 ms, value changes of CI are shown in Figure 1(b). It is found that the sum of CI from SSM and FMM gets the highest value when T2-star is about 48 ms. In* in vivo* cases, *W* of reference signal is also set as 80% of maximum intensity, and optimal T2-star is automatically determined by maximizing sum of CI from SSM and FMM.

### 2.4. Materials

The optimal number of echoes is determined as 12 and its effect in image quality has been discussed [25]. All MR imaging was done on a 3 Tesla Scanner system (Siemens) with GRE sequence (FA: 15 degree; FOV: 220 mm × 220 mm; acquisition matrix: 384 × 384; slice thickness: 3.0 mm; TR: 200 ms). TE values in our experiment range from 2.61 ms to 38.91 ms with an equal interval of 3.3 ms. Each of these four normal healthy volunteers (aged 22, 23, 25, and 29) is scanned four cross sections with slice gap of 0.9 mm parallel to each other for validating image quality. Totally 16 series of MR images are acquired. With optimization procedure, the optimal T2-star values of the 1st volunteer are 54 ms, 56 ms, 55 ms, and 52 ms; of the 2nd volunteer are 46 ms, 47 ms, 47 ms, and 45 ms; of the 3rd volunteer are 46 ms, 47 ms, 47 ms, and 46 ms; and of the 4th volunteer are 49 ms, 48 ms, 48 ms, and 46 ms. SCM, SSM and FMM are generated corresponding to those optimal parameters for generating reference signals.

## 3. Results

### 3.1. Objective Metrics

**Figure 2:**
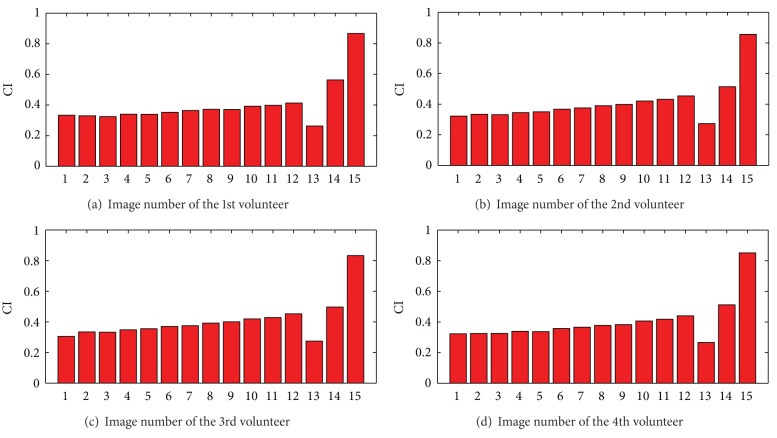
Mean values of CI for these four volunteers. There are original images (1–12), SCM (13), SSM (14), and FMM (15).

With optimal reference signal, CI values of original images, TSM, SSM, and FMM are demonstrated in Figure 2. Each subfigure shows mean value for these four group images. Mean values of CI for original images are stable and increase with ascending TE values. CI values of TSM indexed by 13th drop and CI values of SSM indexed by 14th and FMM indexed by 15th are obviously enhanced to more than 0.50 and 0.85, respectively. Four datasets show similar trend in CI value changes. When TE is 38.91 ms, CI value of original images from (b), (c), and (d) is up to 0.45, and CI value of (a) is slightly higher than 0.4. CI values from SCM drop lower than 0.3, those from SSM increase to be upper than 0.5, and those from FMM highly are improved to around 0.85. All mean values of CI are consistent with very low standard deviation which indicates robustness of acquired data and algorithm.

**Figure 3:**
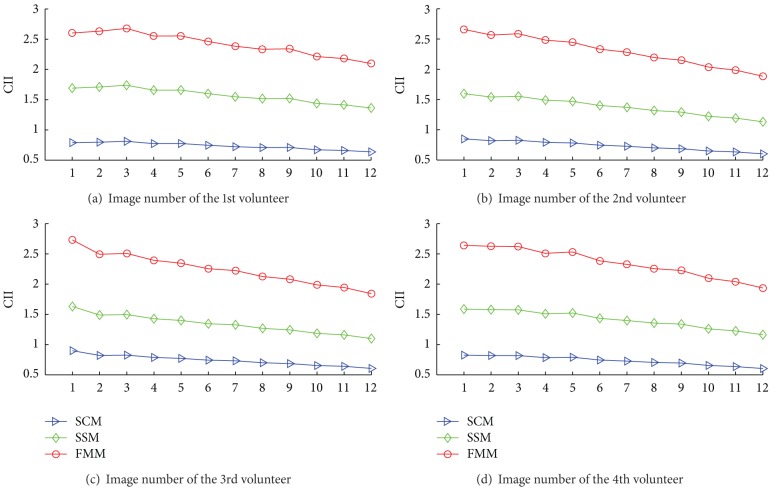
Mean values of CII for these four volunteers. There are CII values by comparing CIs from SCM, SSM and FMM to CIs from these 12 original images.

Quantitative improvement of CI is shown with CII in Figure 3 by comparison with each original image. CII values for SCM, SSM, and FMM are marked with different colors (blue, green, and red, resp.). SCM scores lower than original images. SSM values are higher than original images with average improvement ratio around 57.10% (a), 38.10% (b), 33.92% (c), and 41.22% (d). CII values of FMM are dramatically improved to 241.77% (a), 229.92% (b), 224.34% (c), and 234.86% (d).

### 3.2. Visual Analysis

**Figure 4:**
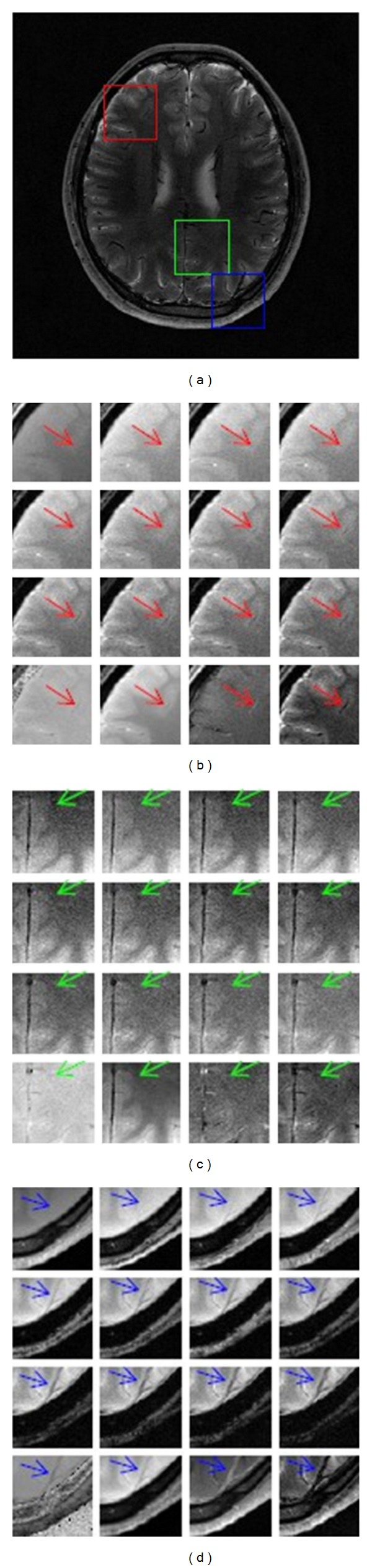
Visual analyses of original images and mappings from the 1st volunteer. (a) It shows three ROIs with different colors. ((b)–(d)) They demonstrate the same ROI from these 12 original images, SCM, SMM, and FMM one by one, respectively.

**Figure 5:**
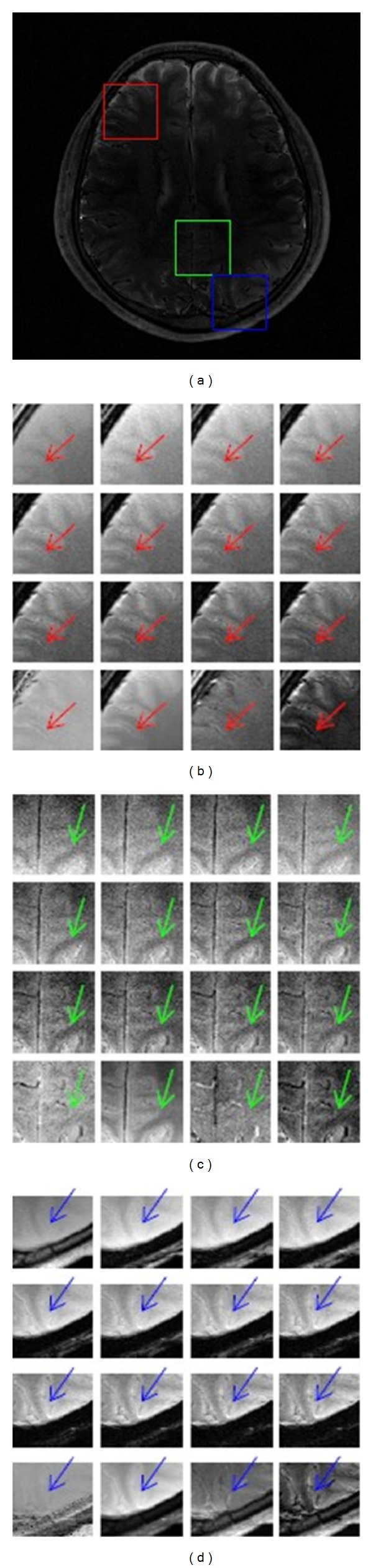
Visual analyses of original images and mappings from the 2nd volunteer. (a) It shows three ROIs with different colors. ((b)–(d)) They demonstrate the same ROI from these 12 original images, SCM, SMM and FMM one by one, respectively.

SSM measures relative signal strength and FMM presents signal fluctuation magnitude with respect to the reference signal. Figures 4 and 5 demonstrate fine structures in original images, T2-star mapping, SCM, SSM and FMM. Three ROIs are in red, green and blue squares. Subfigure (a) shows FMM, and subfigure (b) to (d) show these three ROIs of 12 original images and these 4 mappings.

In Figures 4 and 5, the arrow-directed area becomes more and more obvious in original images when TE increases. T2-star mapping magnifies noise effect and introduces outliers and image contrast is suppressed. SCM is smoothed with restricted noise level and image contrast is consequently decreased. In SSM and FMM, veins are distinguished from soft tissues with clear border. In Figure 4(d), pixel values in veins in SSM are higher than those in surrounding soft tissues which are useful for morphological evaluation, while pixel values in FMM are darker which show lower sensitivity to the reference signal. Whether higher or lower than tissues around, veins in SSM and FMM get better tissue contrast than viens in original images, T2-star mapping, and SCM.

## 4. Discussion and Conclusion

Mapping extraction plays an important role in medical analysis and clinical diagnosis. Q-MRI exploits imaging sequences to extract distribution mappings for absolute biophysical parameters on a pixel-by-pixel basis. TSM in dynamic MRI with contrast agent calculates MSE between any signal temporal responses and a reference ROI temporal response. SCM replaces the signal response to time course in TSM with imaging parameter TE changes in multiecho T2-star weighted MRI without contrast agent involved. Since different tissues are with different sensitivities to reference signals and imaging parameter changes, an improved algorithm is proposed by adding a signal fluctuation index into the theory of SCM and obtains two mappings. Meanwhile, the proposed algorithm is adaptive to generate optimal reference signal by maximizing the sum of contrast indexes from SSM and FMM without manual interaction, since manual delineation of ROI may result in errors and uncertainties.

Image contrast is evaluated from CI and CII. Higher contrast of ROI is useful in distinguishing our focus from its surroundings; even the ROIs are with minor structures, such as veins. From objective analysis, under the same imaging parameters, original images get better contrast at 38.91 ms. SSM and FMM get higher contrast improvement. The enhancement ratio of SSM is 57.10%, 51.39%, 33.92%, and 41.22%, and the enhancement ratio of FMM is 141.77%, 129.92%, 124.34%, and 134.86%. Visual analysis validates the contrast improvement. In both Figures 4 and 5, contrast of ROIs becomes more and more obvious in original images when TE increases. T2-star mapping reduces image quality for magnification of noisy outliers. SCM softens the minor textures with suppressed noise level. SSM and FMM better distinguish soft tissues from surrounding tissues than these original images, T2-star mapping, and SCM.

CI is adopted in this paper to evaluate image quality and search for proper reference signal. That is because CI is a commonly used nonreference metrics, particularly when derived mappings illustrate different meanings and different pixel intensity ranges from conventional medical images. In these clinical cases, SSM and FMM have demonstrated better performance than T2-star mapping and SCM in enhancing image contrast from quantitative evaluation and visual analysis. Using similarity mapping techniques, image quality is improved with suppressed noise. Partial volume effect, intensity inhomogeneity and artifacts are prevalent problems in MR images, but our method is not proper to deal with these problems. Meanwhile, by experiments, we found there was no need to denoise original images, and also there was no need to correct bias field. In addition, our method can be straightly applied to other MRI sequences with one imaging parameter change, such as T1 weighted and T2 weighted MRI, or extended to other temporal image sequences acquired from functional MRI, CT, and PET.

*In vivo* experiments validate the capacity of SSM and FMM from the proposed algorithm in enhancing image contrast and morphological evaluation. These extracted mappings can be further applied in information fusion, signal investigation, tissue segmentation, and medical analysis.
